# Supplementary material for: Chimeric deubiquitinase engineering reveals structural basis for specific inhibition of the mitophagy regulator USP30
Source: Nat Struct Mol Biol. 2025 May 5;32(9):1776–86. doi: 10.1038/s41594-025-01534-4 (PMC12440824; doi:10.1038/s41594-025-01534-4)

nature structural & molecular biology

Supplementary information https://doi.org/10.1038/s41594-025-01534-4

Chimeric deubiquitinase engineering reveals structural basis for specific inhibition of the mitophagy regulator USP30

In the format provided by the authors and unedited

Table of Contents

Supplementary Tables 1 to 3 .... p. 3
Protein sequences .... p. 6
Supplementary Methods .... p. 8
NMR spectra of compounds .... p. 12

Supplementary Table 1. Crystal structures of human USP family DUB catalytic domains.

USP	Zn²⁺ coordination at tip of fingers subdomain	PDB accession codes of apo structures	PDB accession codes of Ub-bound structures	PDB accession codes of Inhibitor-bound structures	Comments
USP1	yes	7AYO*	7 AY2*	7ZH4# (by Cryo-EM)	*in complex with UAF1<br>#with Ub-conjugated to FANCD2
USP2	yes		2HD5, 2IBI, 3NHE, 5XU8*, 5XVE, (+ with Ub variants)		*in complex with Ub and 6-thioguanine
USP4	yes	2Y6E*			*insertions removed by limited proteolysis
USP5	**no**		3IHP*		*full-length
USP7	**no**	1NB8, 2F1Z, 4M5W, 4M5X, 5FWI, 5J7T	1NBF, 5JTJ, 5JTV	5N9R, 5N9T, 5NGE, 5NGF, 5UQV, 5UQX, 5VS6, 5VSB, 5VSK, 5WHC, 6F5H	
USP8	yes	2GFO	3N3K*		*with ubiquitin variant
USP9X	yes	5WCH*			*with surface entropy reducing mutations
USP11	yes		8OYP*		*with Ub-GGG, insertion replaced by RDFrzS tag
USP12	yes	5K1A*, 5K1B*, 5K1C*#, 5K16	5L8W*		*in complex with UAF1<br>#in complex with WDR20
USP14	**no**	2AYN	2AYO, 5GJQ*	6IIN, 6IIM, 6IIL, 6IIK	*Cryo-EM structure of 26S proteasome
USP15	yes	6GHA	6ML1*, 6CPM*, 6CRN*	6GH9	*with ubiquitin variant
USP21	yes		2Y5B, 3I3T, 3MTN*		*with ubiquitin variant
USP25	**no**	5O71, 6H4J, 6HEL			
USP28	**no**	6H4I, 6HEH*, 6HEJ, 8P19*	6H4H, 6HEI*, 6HEK	8HJE*, 7TUO*, 8P14*, 8P1P*, 8P1Q*	*insertions removed
USP30	yes		5OHK#, 5OHN#, 9F6G*	9F19*, 8D0A+, 8D1T+	#insertions removed and with mutations<br>*chimeric construct<br>+ with Fab
USP34	yes	7W3R	7W3U		
USP35	yes		5TXK*		*insertions removed
USP36	yes		8BS9, (8BS3*)		*with Fubi-PA
USP46	yes	6JLQ*#	5CVO*, 5CVN*, 5CVM, 5L8H		*in complex with UAF1<br>#in complex with WDR20
CYLD	**no**	2VHF			

Supplementary Table 2. Biochemical and biophysical characterization of USP30 constructs.

Construct	Catalytic efficiency (x10^3 M^{-1}s^{-1})	Apo USP30 stability (avg. T_m in °C)	Compound inhibitory potency (IC$_{50}$ in nM)		Change in stability upon Inhibitor binding (avg. ΔT_m in °C)		USP30 stability in presence of compound (avg. T_m in °C)	
			Cmpd39	NK036	Cmpd39	NK036	Cmpd39	NK036
c1	213.9 ± 2.4	51.1	0.8 ± 0.04	7.4 ± 0.3	7.8	7.2	58.9	58.3
ch1	655.9 ± 17.6	46.5	0.7 ± 0.04	4.8 ± 1.7	9.6	8.7	56.1	55.2
ch2	221.8 ± 3.6	50.7	0.5 ± 0.06	5.5 ± 0.6	8.4	7.8	59.1	58.5
ch3	195.5 ± 2.4	54.9	0.3 ± 0.03	4.1 ± 0.3	6.6	6.5	61.5	61.4
ch4	5.8 ± 0.0	51.5	-	-	8.8	7.7	60.3	59.2
c2	11.3 ± 0.3	50.7	-	-	7.8	7.5	58.5	58.2
ch3 F453Y	62.7 ± 0.7	56.4	531 ± 99	632 ± 119	2.1	1.8	58.5	58.2
ch3 L328F	14.2 ± 0.2	57.0	-	-	1.5	1.2	58.5	58.2
ch3 I154A	-	55.2	79 ± 11	76 ± 8	3.8	3.8	59.0	59.0
ch3 F157A	-	54.9	51 ± 8	97 ± 8	3.9	3.6	58.8	58.5
ch3 A162S	-	55.8	63 ± 9	70 ± 5	4.2	3.9	60.0	59.7

Supplementary Table 3. Statistics of individual datasets used for multi-crystal averaging.

	USP30 + NK036 (crystal 1)	USP30 + NK036 (crystal 2)	USP30 + NK036 (crystal 3)	USP30 + NK036 (blended from crystals 1+2+3)
**Data collection**				
Beamline	ESRF ID30A-3	ESRF ID30A-3	ESRF ID30A-3	ESRF ID30A-3
Wavelength	0.9677 Å	0.9677 Å	0.9677 Å	0.9677 Å
Space group	$P\,2_1\,2\,2_1$	$P\,2_1\,2\,2_1$	$P\,2_1\,2\,2_1$	$P\,2_1\,2\,2_1$
Cell dimensions				
a, b, c (Å)	55.90, 73.64, 201.66	55.96, 73.15, 200.37	55.92, 74.14, 201.67	55.83, 73.84, 201.05
α, β, γ (°)	90, 90, 90	90, 90, 90	90, 90, 90	90, 90, 90
Anisotropy correction	yes	yes	yes	yes
Observed reflections	59,699	49,129	62,471	214,419
Unique reflections	12,735	11,139	12,592	15,438
Resolution (Å)	48.89 – 2.95<br>(3.23 – 2.95)	68.71 – 3.03<br>(3.37 – 3.03)	53.89 – 3.01<br>(3.31 – 3.01)	59.52 – 2.75<br>(3.23 – 2.75)
Ellipsoidal resolution limits (Å) [direction]	2.95 [a*]<br>3.81 [b*]<br>2.95 [c*]	2.97 [a*]<br>3.67 [b*]<br>3.28 [c*]	2.95 [a*]<br>3.84 [b*]<br>3.01 [c*]	2.89 [a*]<br>3.53 [b*]<br>2.75 [c*]
R_{merge}	0.131 (1.274)	0.120 (0.729)	0.134 (1.079)	0.166 (1.536)
R_{meas}	0.147 (1.397)	0.137 (0.888)	0.150 (1.193)	0.173 (1.595)
$I/\sigma(I)$	7.2 (1.4)	6.6 (1.7)	7.3 (1.4)	9.6 (1.9)
$CC_{1/2}$	0.955 (0.611)	0.997 (0.526)	0.959 (0.631)	0.998 (0.730)
Spherical completeness (%)	69.7 (20.2)	66.8 (14.9)	72.8 (25.6)	68.7 (26.2)
Ellipsoidal completeness (%)	89.3 (56.6)	85.2 (48.8)	90.7 (62.1)	91.9 (69.0)
Redundancy	4.7 (5.4)	4.4 (3.2)	5.0 (5.6)	13.9 (13.9)
Wilson B (Å^2) [direction]	85 [a*]<br>166 [b*]<br>77 [c*]	84 [a*]<br>177 [b*]<br>56 [c*]	77 [a*]<br>158 [b*]<br>70 [c*]	82 [a*]<br>161 [b*]<br>71 [c*]

Protein sequences

Construct c1 – USP30Δ
boundaries: **64-178; GSGS; 217-357; SNA; 432-502**
mutations: **F348D, M350S, I353E**
KGLVPGLVNLGNTCFMNSLLQGLSACPAFIRWLEEFTSQYSRDQKEPPSHQYLSLTLLHL
LKALSCQEVTDDEVLDASCLLDVLRMYRWQISSFEEQDAHELFHVITSSLEDERDGSGS
HWKSQHPFHGRLTSNMVCKHCEHQSPVRFDTFDSLSLSIPAATWGHPLTLDHCLHHFIS
SESVRDVVCDNCTKIEAKGTLNGEKVEHQRTTFVKQLKLGKLPQCLCIHLQRLSWSSHG
TPLKRHEHVQFNE**D**L**S**MD**E**YKYHSNASTYLFRLMAVVVHHGDMHSGHFVTYRRSPPS
ARNPLSTSNQWLWVSDDTVRKASLQEVLSSSAYLLFYERV

Construct ch1 – USP30 (USP7)
boundaries: **64-178; GSGS; 217-225 (326-348) 249-275 (370-398) 318-357; SNA; 432-502**
mutations: **F348D, M350S, I353E**
KGLVPGLVNLGNTCFMNSLLQGLSACPAFIRWLEEFTSQYSRDQKEPPSHQYLSLTLLHL
LKALSCQEVTDDEVLDASCLLDVLRMYRWQISSFEEQDAHELFHVITSSLEDERDGSGS
HWKSQHPFHGKMVSYIQCKEVDYRSDRREDYYDSLSLSIPAATWGHPLTLDHCLHHFIS
VEQLDGDNKYDAGEHGLQEAEKGVKFLTLPQCLCIHLQRLSWSSHGTPLKRHEHVQFN
E**D**L**S**MD**E**YKYHSNASTYLFRLMAVVVHHGDMHSGHFVTYRRSPPSARNPLSTSNQWL
WVSDDTVRKASLQEVLSSSAYLLFYERV

Construct ch2 – USP30 (USP14)
boundaries: **64-178; GSGS; 217-224 (248-272) 249-275 (295-320) 318-357; SNA; 432-502**
mutations: **F348D, M350S, I353E**
KGLVPGLVNLGNTCFMNSLLQGLSACPAFIRWLEEFTSQYSRDQKEPPSHQYLSLTLLHL
LKALSCQEVTDDEVLDASCLLDVLRMYRWQISSFEEQDAHELFHVITSSLEDERDGSGS
HWKSQHPFGVEFETTMKCTESEEEEVTKGKENQDSLSLSIPAATWGHPLTLDHCLHHFIS
QEEITKQSPTLQRNALYIKSSKISRLPQCLCIHLQRLSWSSHGTPLKRHEHVQFNE**D**L**S**MD
EYKYHSNASTYLFRLMAVVVHHGDMHSGHFVTYRRSPPSARNPLSTSNQWLWVSDDT
VRKASLQEVLSSSAYLLFYERV

Construct ch3 – USP30 (USP14) [USP35]
boundaries: **64-178; GSGS; 217-224 (248-272) 249-275 (295-320) 318-348 [833-845] 437-502**
mutations: **F348D**
KGLVPGLVNLGNTCFMNSLLQGLSACPAFIRWLEEFTSQYSRDQKEPPSHQYLSLTLLHL
LKALSCQEVTDDEVLDASCLLDVLRMYRWQISSFEEQDAHELFHVITSSLEDERDGSGS
HWKSQHPFGVEFETTMKCTESEEEEVTKGKENQDSLSLSIPAATWGHPLTLDHCLHHFIS
QEEITKQSPTLQRNALYIKSSKISRLPQCLCIHLQRLSWSSHGTPLKRHEHVQFNE**D**LRLP
LAGGRGQAYRLMAVVVHHGDMHSGHFVTYRRSPPSARNPLSTSNQWLWVSDDTVRK
ASLQEVLSSSAYLLFYERV

Construct ch4 – USP30 (CYLD)
boundaries: **64-178; GSGS; 217-225 (700-713) 250-275 (742-746) 317-357; SNA; 432-502**
mutations: **F348D, M350S, I353E**
KGLVPGLVNLGNTCFMNSLLQGLSACPAFIRWLEEFTSQYSRDQKEPPSHQYLSLTLLHL
LKALSCQEVTDDEVLDASCLLDVLRMYRWQISSFEEQDAHELFHVITSSLEDERDGSGS
HWKSQHPFHLKIRSAGQKVQDCYSLSLSIPAATWGHPLTLDHCLHHFISNLKFALPQCLC
IHLQRLSWSSHGTPLKRHEHVQFNE**D**L**S**MD**E**YKYHSNASTYLFRLMAVVVHHGDMHS
GHFVTYRRSPPSARNPLSTSNQWLWVSDDTVRKASLQEVLSSSAYLLFYERV

Construct c2 – USP30 (GS)
boundaries: **64-178; GSGS; 217-225; GSGS; 250-276; GSGSGS; 317-357; SNA; 432-502**
mutations: **F348D, M350S, I353E**
KGLVPGLVNLGNTCFMNSLLQGLSACPAFIRWLEEFTSQYSRDQKEPPSHQYLSLTLLHL
LKALSCQEVTDDEVLDASCLLDVLRMYRWQISSFEEQDAHELFHVITSSLEDERDGSGS
HWKSQHPFHGSGSSLSLSIPAATWGHPLTLDHCLHHFISGSGSGSLPQCLCIHLQRLSWSS
HGTPLKRHEHVQFNE**D**L**S**MD**E**YKYHSNASTYLFRLMAVVVHHGDMHSGHFVTYRRSP
PSARNPLSTSNQWLWVSDDTVRKASLQEVLSSSAYLLFYERV

Supplementary Methods

General notes on synthetic methods

The chemicals and solvents used for this work were purchased from companies such as Activate Scientific, BLDpharm, Fisher Scientific, Sigma-Aldrich, TCI and VWR and used without further purification. Solvents used for the synthesis were named as follows. EA: ethyl acetate, DCM: dichloromethane, MeOH: methanol, DMF: dimethylformamide, DMSO: dimethyl sulfoxide, THF: tetrahydrofuran, ACN: acetonitrile, H_2O: water, EtOH: ethanol. Silica gel aluminum plates (silica gel 60 F254, Merck) were used for thin-layer chromatography and the detection was carried out using UV light at 254 and 360 nm. A Pure C-850 FlashPrep system (Büchi) was used for automated column chromatographic purification of the final product using a VP125/21 Nucleodur C18 Gravity column (21 x 125 mm; 5 µm, Macherey Nagel) by using a 0-100% gradient of H_2O + 0.1% TFA to ACN + 0.1% TFA over 70 min.

A 1200 Infinity series HPLC system (Agilent Technologies) with a ZORBAX Eclipse XDB column (C18 80 Å; 4.6 x 150 mm; 5 µm, Agilent Technologies) was used for low resolution LC-MS analysis. For high resolution mass spectrometry (HRMS) an LTQ Orbitrap Fourier transform mass spectrometer (Thermo Fisher Scientific) coupled to an HPLC instrument from the same company (Hypersil Gold: 1 x 50 mm; 1.9 µm) was used. H_2O + 0.1% formic acid and ACN + 0.1% formic acid were used as eluents. The ionization mode was ESI (Electrospray ionization) with a source voltage of 3.8 kV.

The following devices from Bruker were used to record NMR spectra: AV 500 Avance III HD (500 MHz for ^{1}H and 125 MHz for ^{13}C-NMR), AV 600 Avance III HD (600 MHz for ^{1}H and 151 MHz for ^{13}C NMR) and AV 700 Avance III HD (700 MHz for ^{1}H and 176 MHz for ^{13}C NMR). The chemical shifts of all spectra are specified in ppm and the coupling constants *J* are given in Hertz (Hz). Peaks of deuterated solvents were used as internal standards for ^{1}H / ^{13}C data (DMSO-d_6: δ = 2.50 ppm / 39.52 ppm). The multiplicities of the signals are abbreviated as follows: s (singlet), d (doublet), dd (doublet of doublets), ddd (doublet of doublets of doublets), t (triplet), q (quartet) and m (multiplet).

Any syntheses or derivatizations of patented compounds were used for research purposes only.

Synthesis of (*S*)-4-fluoro-*N*-(1-((4-(*N*-(1-hydroxy-2-methylpropan-2-yl)sulfamoyl)phenyl)-amino)-1-oxo-3-phenylpropan-2-yl)benzamide (NK036)

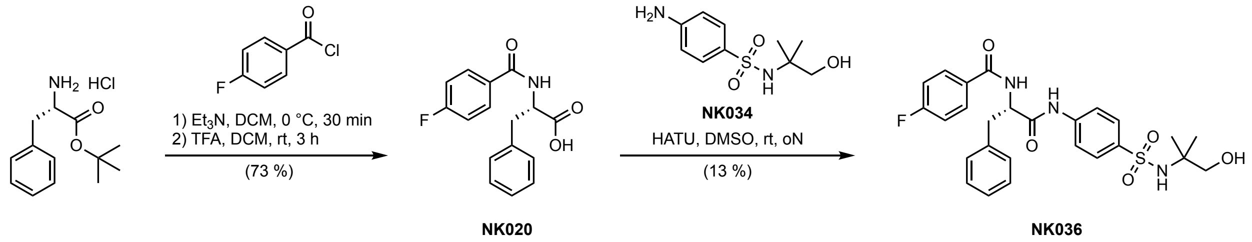

The synthesis was based on previously described methods[32] for the synthesis of compound 39, with adjustments for the synthesis of (4-fluorobenzoyl)-L-phenylalanine (NK020) based on the synthesis of a related compound.[80] A different synthesis route of the title compound NK036 (I-137) was published elsewhere.[34]

Synthesis of (4-fluorobenzoyl)-L-phenylalanine (NK020)

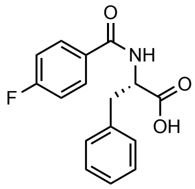

Step 1:

tert-Butyl L-phenylalaninate hydrochloride (2.8 g, 11 mmol, 1.0 eq) and triethylamine (2.8 g, 27.5 mmol, 2.5 eq) were dissolved in DCM (30 mL) and cooled to 0°C. 4-Fluorobenzoyl chloride (2.1 g, 13.2 mmol, 1.2 eq) was added dropwise while stirring. The reaction was stirred for 30 min at 0°C until completion. The reaction was quenched by the addition of water (50 mL). The aqueous layer was extracted with DCM (2 x 50 mL), and the combined organic phases were washed with brine (2 x 50 mL) and dried over MgSO4. The solvent was evaporated under reduced pressure to obtain the product *tert*-butyl (4-fluorobenzoyl)-L-phenylalaninate (NK019) as a yellow oil. The crude product was used for the next step without further purification.

Step 2:

tert-Butyl (4-fluorobenzoyl)-L-phenylalaninate (3.7 g, 11 mmol) was dissolved in HCl/dioxan (4 M, 20 mL) and stirred at rt for 3 h. After completion, the solvents were evaporated under reduced pressure to obtain the product (4-fluorobenzoyl)-L-phenylalanine (NK020) as an off-white powder (2.9 g, 10.1 mmol, 73 % over two steps).

^{1}H NMR (600 MHz, DMSO) δ (ppm): 12.81 (s, 1H), 8.77 (d, *J* = 8.2 Hz, 1H), 7.89 (dd, *J* = 8.8, 5.6 Hz, 2H), 7.34 – 7.31 (m, 2H), 7.31 – 7.24 (m, 4H), 7.20 – 7.15 (m, 1H), 4.65 (ddd, *J* = 10.7, 8.1, 4.4 Hz, 1H), 3.22 (dd, *J* = 13.8, 4.4 Hz, 1H), 3.08 (dd, *J* = 13.9, 10.7 Hz, 1H).

^{13}C NMR (151 MHz, DMSO) δ (ppm): 173.25, 165.42, 164.03 (d, *J* = 248.7 Hz), 138.21, 130.42 (d, *J* = 2.8 Hz), 130.08 (d, *J* = 8.9 Hz), 129.10, 128.26, 126.43, 115.25 (d, *J* = 21.7 Hz), 54.35, 36.34.

HRMS m/z for $C_{16}H_{15}FNO_3^+$ ($[M+H]^+$) calculated: 288.1031, found: 288.1029.

Synthesis of (*S*)-4-fluoro-*N*-(1-((4-(*N*-(1-hydroxy-2-methylpropan-2-yl)sulfamoyl)phenyl)-amino)-1-oxo-3-phenylpropan-2-yl)benzamide (NK036)

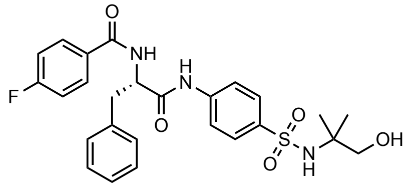

4-Fluorbenzoyl-L-phenylalanine (NK020) (70.6 mg, 0.25 mmol, 1.2 eq) was dissolved in dry DMSO (2 mL). To that solution was added HATU (155.6 mg, 0.41 mmol, 2 eq) and the resulting mixture was stirred for 30 minutes at rt. 4-Amino-*N*-(1-hydroxy-2-methylpropan-2-yl)benzenesulfon-amide (NK034) (50 mg, 0.2 mmol, 1.0 eq) was added and the reaction stirred overnight. The resulting solution was then directly purified by reverse phase column chromatography. The product NK036 was obtained as a white powder (13.2 mg, 0.03 mmol, 13 %).

^{1}H NMR (700 MHz, DMSO-d_6) δ (ppm): 10.58 (s, 1H), 8.86 (d, *J* = 7.9 Hz, 1H), 7.92 – 7.89 (m, 2H), 7.77 (s, 4H), 7.41 (d, *J* = 7.1 Hz, 2H), 7.32 – 7.26 (m, 4H), 7.21 – 7.16 (m, 2H), 4.84 (ddd, *J* = 10.4, 7.9, 4.8 Hz, 1H), 3.17 (s, 2H), 3.16 – 3.08 (m, 2H), 1.00 (s, 6H).

^{13}C NMR (176 MHz, DMSO-d_6) δ (ppm): 171.08, 165.56, 163.97 (d, J = 248.8 Hz), 141.82, 138.59, 137.97, 130.22 (d, J = 2.9 Hz), 130.17 (d, J = 9.0 Hz), 129.17, 128.12, 127.40, 126.41, 118.87, 115.16 (d, J = 21.7 Hz), 68.93, 56.81, 56.06, 36.90, 24.02.

HRMS m/z for $C_{26}H_{29}FN_3O_5S^+$ ($[M+H]^+$) calculated: 514.1806, found: 514.1811.

Synthesis of 4-amino-*N*-(1-hydroxy-2-methylpropan-2-yl)benzenesulfonamide (NK034)

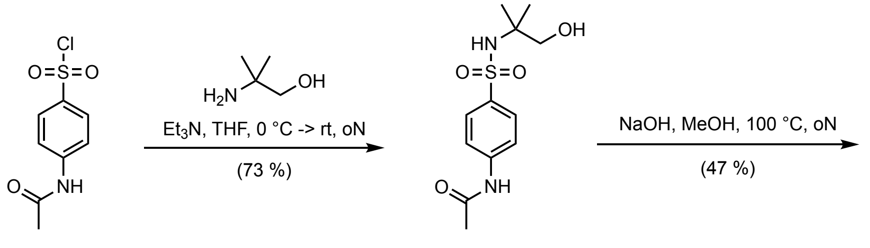

NK027　　NK034

Synthesis of *N*-(4-(*N*-(1-hydroxy-2-methylpropan-2-yl)sulfamoyl)phenyl)acetamide (NK027)

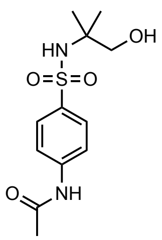

To a solution of 4-acetamidobenzenesulfonyl chloride (5 g, 21.4 mmol, 1.0 eq) in dry THF (30 mL) was added dropwise triethylamine (4.3 g, 42.8 mmol, 2 eq) and 2-amino-2-methyl-1-propanol (3.8 g, 42.8 mmol, 2 eq) at 0°C while stirring. The reaction was then warmed to rt and stirred over night until completion. The mixture was then poured into a mixture of ice and water (50 mL). The product was extracted with EA (3 x 50 mL) and the combined organic layers were washed with saturated NH_4Cl solution (2 x 100 mL), water (1 x 100 mL) and brine (1 x 100 mL). The organic layer was dried over anhydrous $MgSO_4$ after which the solvents were removed under reduced pressure. The product *N*-(4-(*N*-(1-hydroxy-2-methylpropan-2-yl)sulfamoyl)phenyl)-acetamide (NK027) was obtained as a white solid (4.5 g, 15.7 mmol, 73 %).

^{1}H NMR (500 MHz, DMSO-d_6) δ (ppm): 10.28 (s, 1H), 7.81 – 7.65 (m, 4H), 7.17 (s, 1H), 4.73 (t, J = 5.9 Hz, 1H), 3.16 (d, J = 6.0 Hz, 2H), 2.08 (s, 3H), 0.98 (s, 6H).

^{13}C NMR (126 MHz, DMSO-d_6) δ (ppm): 168.96, 142.29, 138.13, 127.40, 118.43, 68.96, 56.81, 24.15, 24.03.

LCMS m/z for $C_{12}H_{19}N_2O_4S^+$ ($[M+H]^+$) calculated: 287.1, found: 287.2.

Synthesis of 4-amino-*N*-(1-hydroxy-2-methylpropan-2-yl)benzenesulfonamide (NK034)

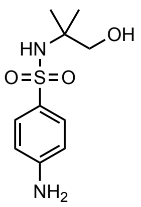

To a solution of *N*-(4-(*N*-(1-hydroxy-2-methylpropan-2-yl)sulfamoyl)phenyl) acetamide (NK027) (520 mg, 1.82 mmol) in methanol (4 mL) was added aq. NaOH (5 M, 3 mL). The reaction mixture was stirred for 100°C over night. After completion, the reaction was cooled to room temperature and diluted with water (10 mL). The organic solvent was removed under reduced pressure. The aqueous solution was then neutralized with aq. HCl (2 M) to pH 7-8, after which the product was extracted with EA (2 x 50 mL). The combined organic layers were dried over $MgSO_4$, and the solvent was removed under reduced pressure which yielded the product 4-amino-*N*-(1-hydroxy-2-methylpropan-2-yl)benzenesulfonamide (NK034) as a white solid (210 mg, 0.83 mmol) which was directly used for the next step without further purification.

HRMS m/z for $C_{10}H_{17}N_2O_3S^+$ ($[M+H]^+$) calculated: 245.0954, found: 245.0953.

Synthesis of (*S*)-*N*-(1-((4-(*N*-(*tert*-Butyl)sulfamoyl)phenyl)amino)-1-oxo-3-phenylpropan-2-yl)-4-fluorobenzamide (compound 39)

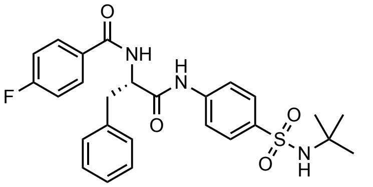

Compound 39 was synthesized using the same synthetic procedure as described above. The analytical data are in full agreement with previously published ^{1}H NMR data.

^{1}H NMR (700 MHz, DMSO-*d*$_6$) δ (ppm): 10.58 (s, 1H), 8.86 (d, *J* = 7.9 Hz, 1H), 7.90 (dd, *J* = 8.7, 5.6 Hz, 2H), 7.77 (s, 4H), 7.42 – 7.40 (m, 3H), 7.29 (q, *J* = 8.1, 7.3 Hz, 4H), 7.18 (t, *J* = 7.4 Hz, 1H), 4.87 – 4.81 (m, 1H), 3.19 – 3.05 (m, 2H), 1.08 (s, 9H).

^{13}C NMR (176 MHz, DMSO-*d*$_6$) δ (ppm): 171.08, 165.55, 163.97 (d, *J* = 248.9 Hz), 141.81, 138.43, 137.96, 130.22 (d, *J* = 2.9 Hz), 130.17 (d, *J* = 9.0 Hz), 129.17, 128.12, 127.44, 126.40, 118.90, 115.16 (d, *J* = 21.8 Hz), 56.06, 53.11, 36.90, 29.73.

HRMS m/z for $C_{26}H_{29}FN_3O_4S^+$ ($[M+H]^+$) calculated: 498.1857, found 498.1850.

NMR spectra of compounds

NK020 ^{1}H NMR

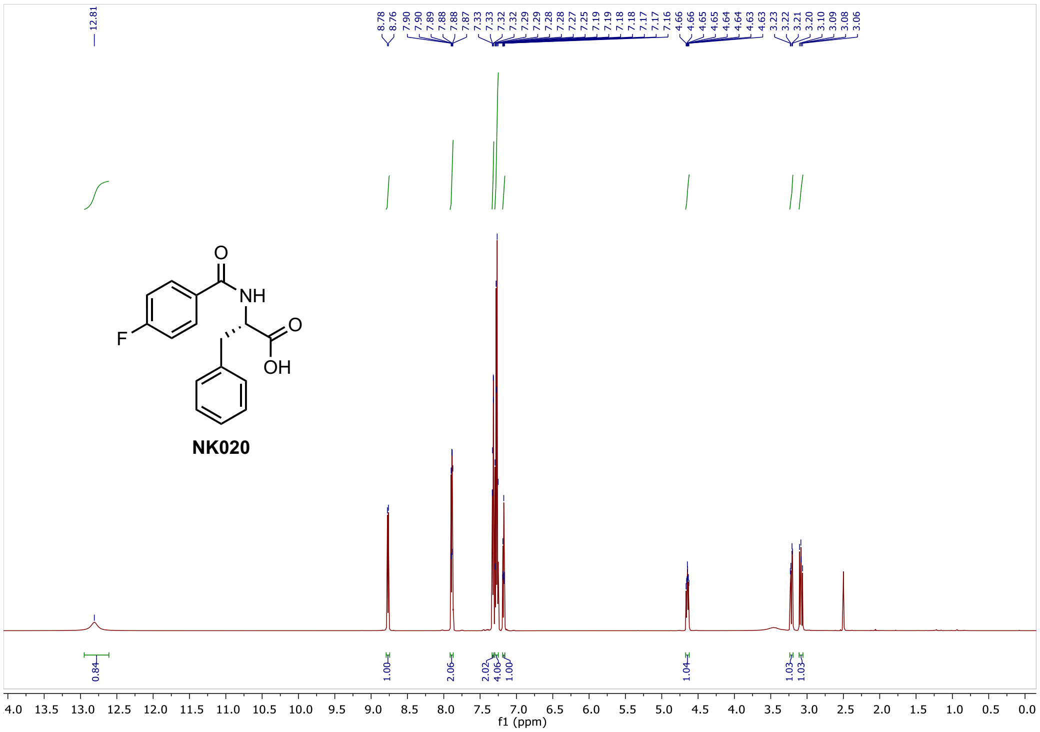

NK020 ^{13}C NMR

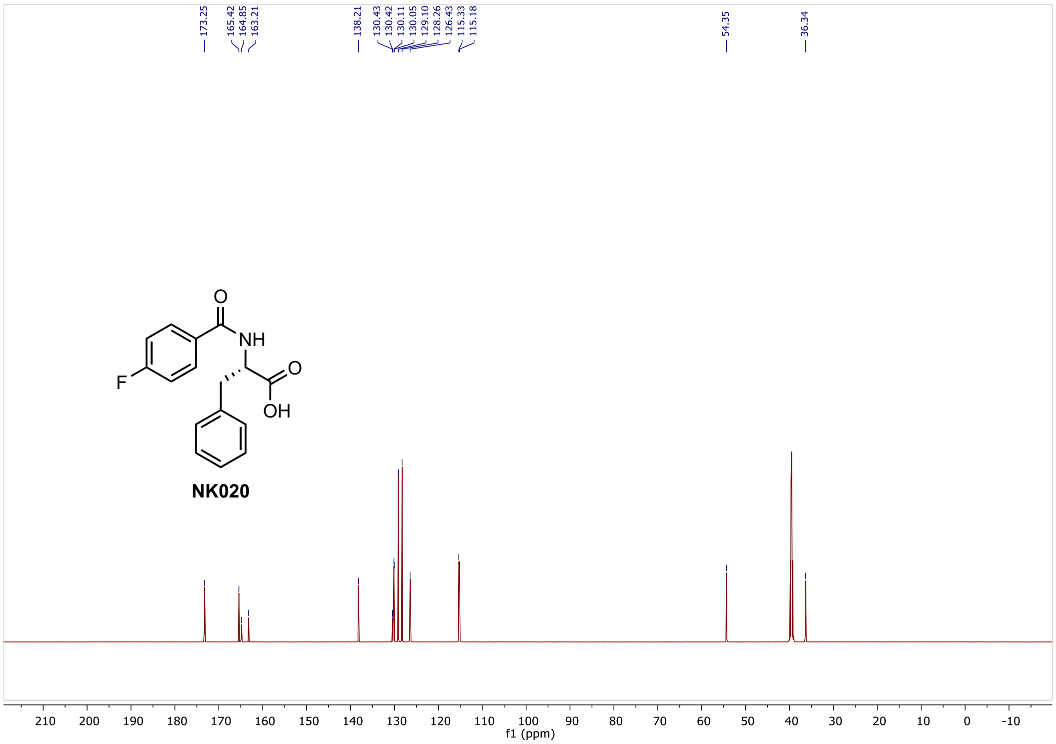

NK036 ^{1}H NMR

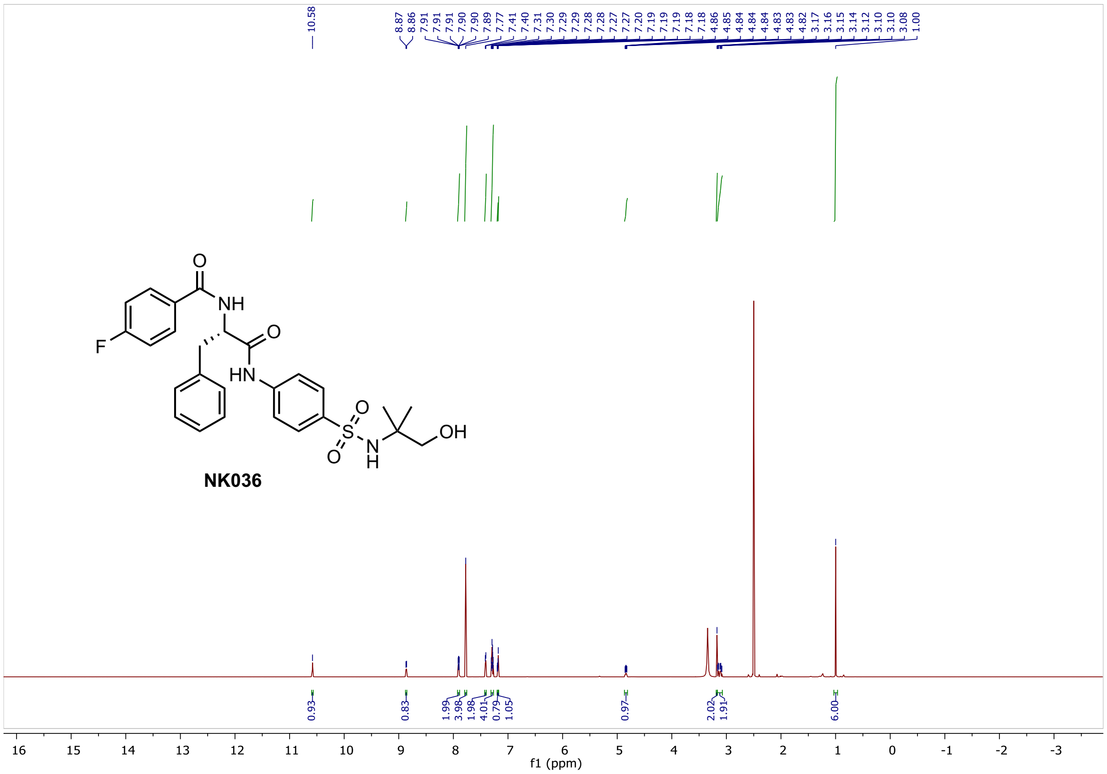

NK036 ^{13}C NMR

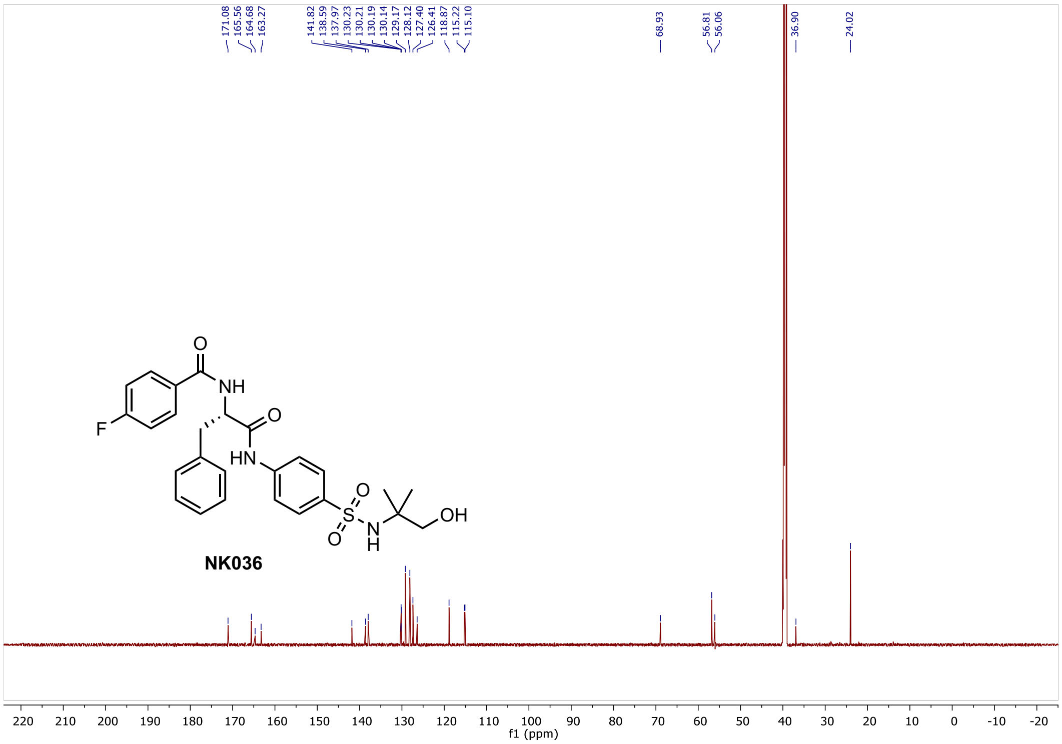

NK027 ^{1}H NMR

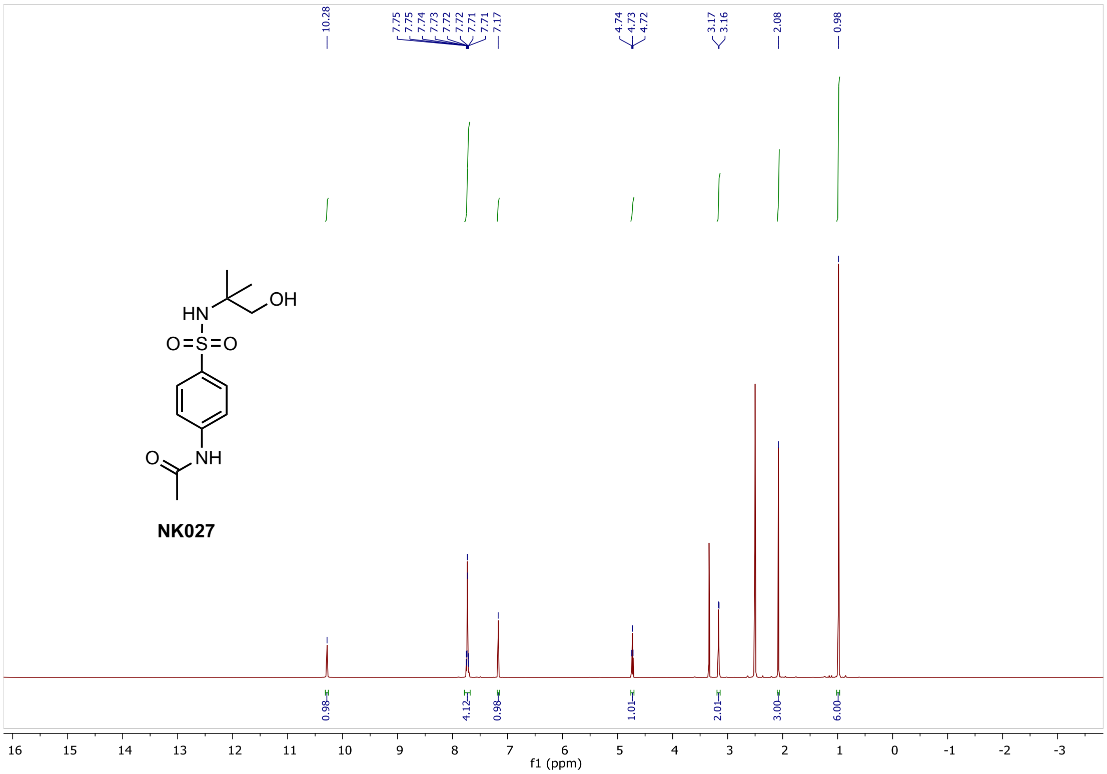

NK027 ^{13}C NMR

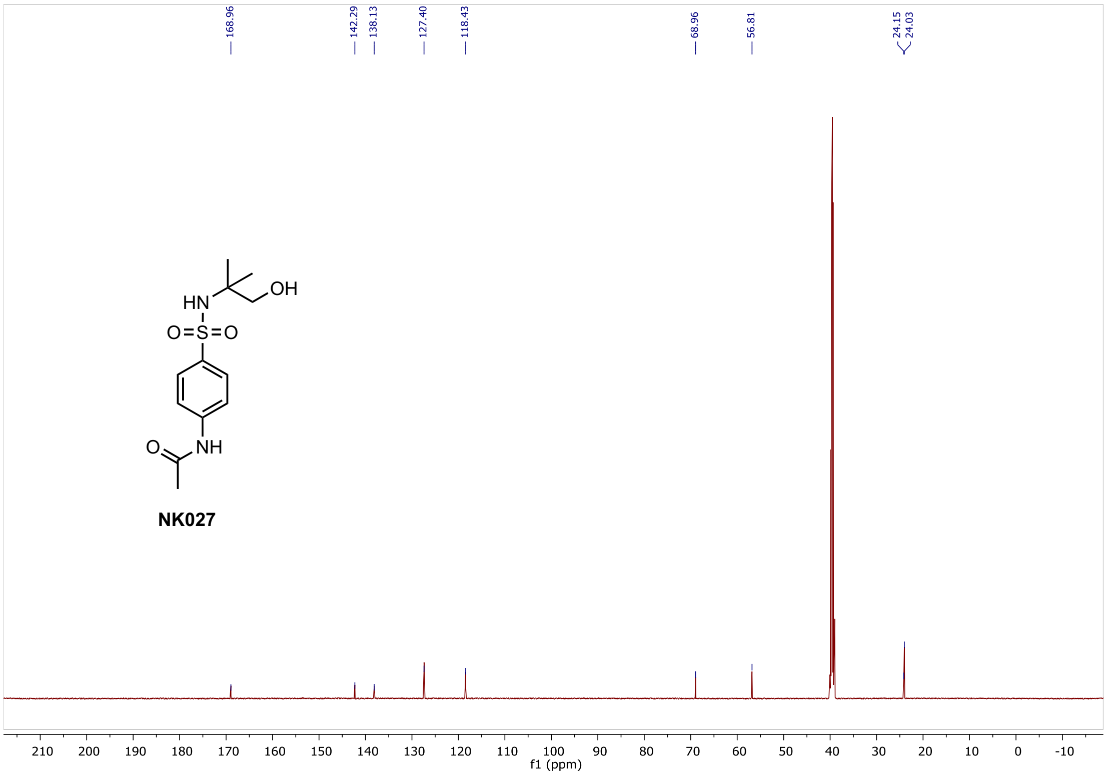

Compound 39 ^{1}H NMR

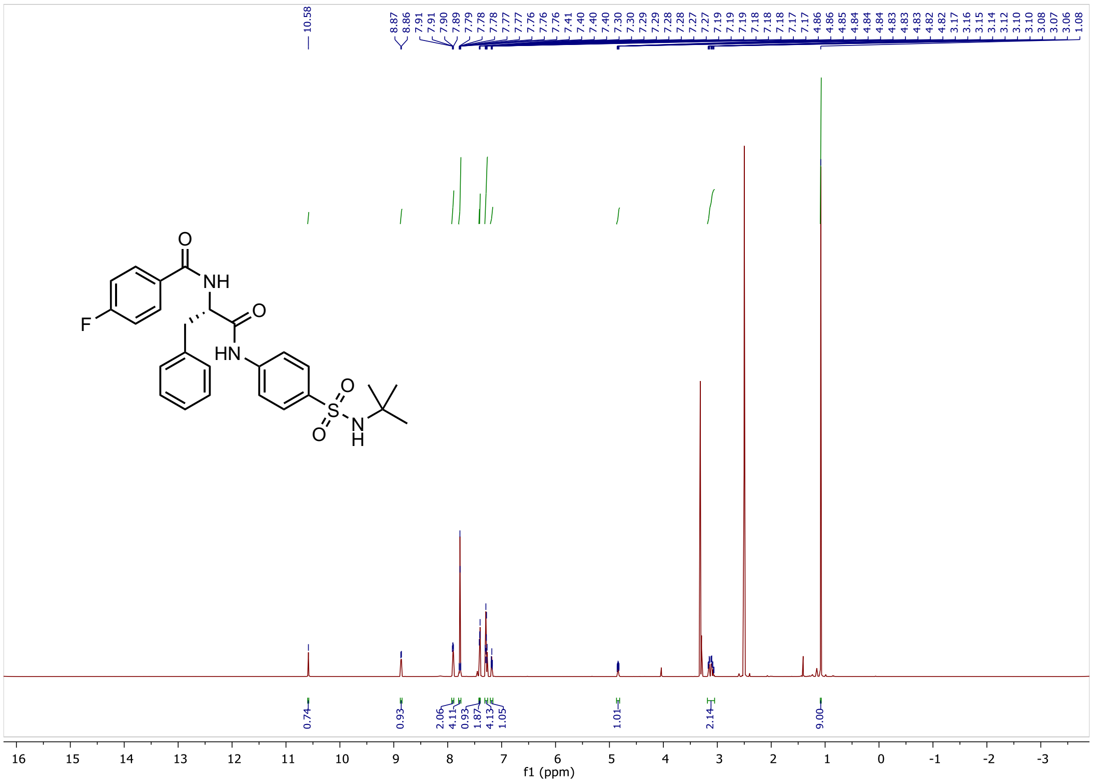

Compound 39 ^{13}C NMR

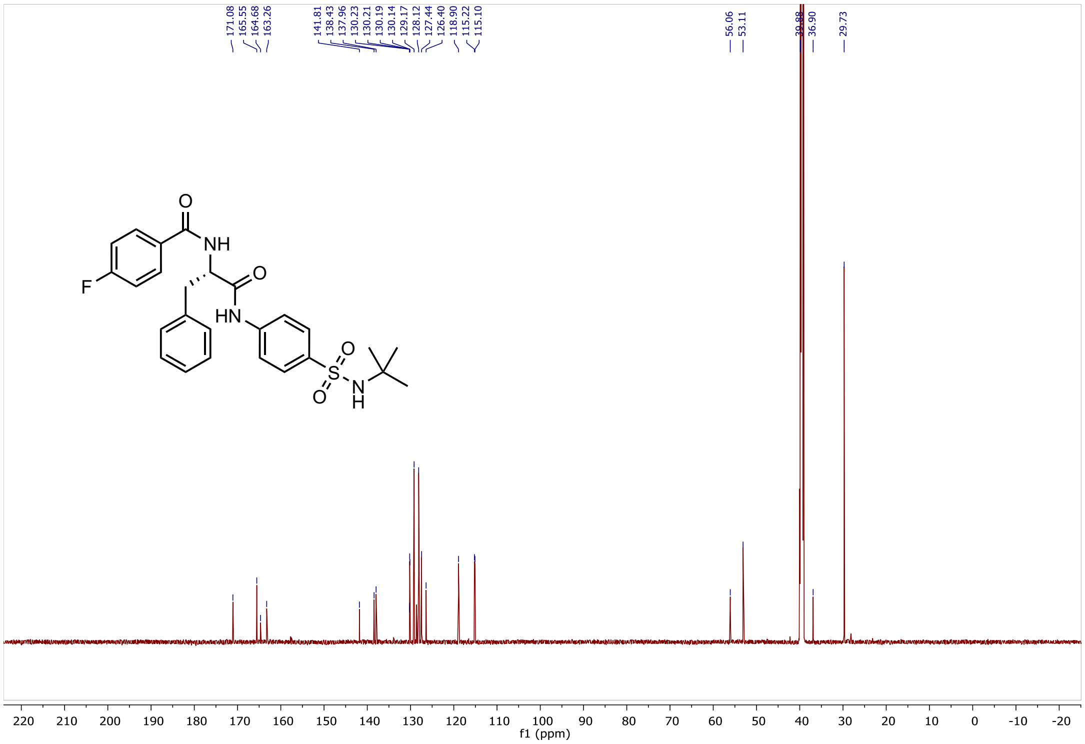

Supplement: Supplementary file 1 — Supplementary Tables 1–3, protein sequences, Methods and NMR spectra of compounds [file 41594_2025_1534_MOESM1_ESM.pdf]
