# Supplementary material for: Chimeric deubiquitinase engineering reveals structural basis for specific inhibition of the mitophagy regulator USP30
Source: Nat Struct Mol Biol. 2025 May 5;32(9):1776–86. doi: 10.1038/s41594-025-01534-4 (PMC12440824; doi:10.1038/s41594-025-01534-4)
Supplement: Supplementary file 2 — Reporting Summary [file 41594_2025_1534_MOESM2_ESM.pdf]

# nature portfolio

Corresponding author(s): Malte Gersch

Last updated by author(s): Feb 25, 2025



# Reporting Summary

Nature Portfolio wishes to improve the reproducibility of the work that we publish. This form provides structure for consistency and transparency in reporting. For further information on Nature Portfolio policies, see our Editorial Policies and the Editorial Policy Checklist.

## Statistics

For all statistical analyses, confirm that the following items are present in the figure legend, table legend, main text, or Methods section.

| n/a | Confirmed | |
|---|---|---|
| ☐ | ☒ | The exact sample size (*n*) for each experimental group/condition, given as a discrete number and unit of measurement |
| ☒ | ☐ | A statement on whether measurements were taken from distinct samples or whether the same sample was measured repeatedly |
| ☒ | ☐ | The statistical test(s) used AND whether they are one- or two-sided<br>*Only common tests should be described solely by name; describe more complex techniques in the Methods section.* |
| ☒ | ☐ | A description of all covariates tested |
| ☒ | ☐ | A description of any assumptions or corrections, such as tests of normality and adjustment for multiple comparisons |
| ☐ | ☒ | A full description of the statistical parameters including central tendency (e.g. means) or other basic estimates (e.g. regression coefficient) AND variation (e.g. standard deviation) or associated estimates of uncertainty (e.g. confidence intervals) |
| ☒ | ☐ | For null hypothesis testing, the test statistic (e.g. *F*, *t*, *r*) with confidence intervals, effect sizes, degrees of freedom and *P* value noted<br>*Give P values as exact values whenever suitable.* |
| ☒ | ☐ | For Bayesian analysis, information on the choice of priors and Markov chain Monte Carlo settings |
| ☒ | ☐ | For hierarchical and complex designs, identification of the appropriate level for tests and full reporting of outcomes |
| ☒ | ☐ | Estimates of effect sizes (e.g. Cohen's *d*, Pearson's *r*), indicating how they were calculated |

*Our web collection on statistics for biologists contains articles on many of the points above.*

## Software and code

Policy information about availability of computer code

| | |
|---|---|
| Data collection | SparkControl (Tecan, V2.3); CFX Maestro (Bio-Rad, V4.1.2433.1219); OpenLab CDS (Agilent, V2.4) |
| Data analysis | Image Lab (Bio-Rad, V2.4.0.03); Prism (GraphPad, V9); Pymol (Schrödinger, V2.5.5); Dials (V3.19.0 and V3.16.0); Aimless (V0.7.15); Staraniso (V3.350); MR Phaser (V2.8.3); Coot (V0.9.8.93); Phenix.Refine (V1.20.1); ORCA (V5.0.4); Alphafold (V2.3.0); eLBOW (V1.20.1) |

For manuscripts utilizing custom algorithms or software that are central to the research but not yet described in published literature, software must be made available to editors and reviewers. We strongly encourage code deposition in a community repository (e.g. GitHub). See the Nature Portfolio guidelines for submitting code & software for further information.

## Data

Policy information about availability of data

All manuscripts must include a data availability statement. This statement should provide the following information, where applicable:

- Accession codes, unique identifiers, or web links for publicly available datasets
- A description of any restrictions on data availability
- For clinical datasets or third party data, please ensure that the statement adheres to our policy

Coordinates and structure factors for the USP30ch3 + NK036 and USP30ch3~Ub-PA crystal structures were deposited with the protein databank (PDB) under accession codes 9F19 and 9F6G, respectively. Coordinates of other structures were obtained from the PDB through accession codes 2AYN, 5N9R, 5OHK, 5OHP, 5TXK, 5UQX, 6GH9, 6IIM, 6IIN, 6VN3, 7ZH4, 8P1Q. Source data (uncropped gels and blots, numerical data) are provided with this paper.

## Research involving human participants, their data, or biological material

Policy information about studies with human participants or human data. See also policy information about sex, gender (identity/presentation), and sexual orientation and race, ethnicity and racism.

| | |
|---|---|
| Reporting on sex and gender | Human research participants were not involved in this study. |
| Reporting on race, ethnicity, or other socially relevant groupings | Human research participants were not involved in this study. |
| Population characteristics | Human research participants were not involved in this study. |
| Recruitment | Human research participants were not involved in this study. |
| Ethics oversight | Human research participants were not involved in this study. |

Note that full information on the approval of the study protocol must also be provided in the manuscript.

# Field-specific reporting

Please select the one below that is the best fit for your research. If you are not sure, read the appropriate sections before making your selection.

☒ Life sciences ☐ Behavioural & social sciences ☐ Ecological, evolutionary & environmental sciences

For a reference copy of the document with all sections, see nature.com/documents/nr-reporting-summary-flat.pdf

# Life sciences study design

All studies must disclose on these points even when the disclosure is negative.

| | |
|---|---|
| Sample size | For quantitative experiments, a sample size of 3 to 6 independent experiments was chosen in line with what is the standard of the field in the molecular biosciences. Sample sizes are given in the figure captions. |
| Data exclusions | Data exclusion occurred in the processing of crystallographic data as implemented in the respective software (e.g. in Phenix.Refine during the scaling of input intensities and subsequent outlier rejection according to expected intensity statistics). These processes took place completely automated as is the default in these programs and without any customization or user input. |
| Replication | All observations were made in at least two independent experiments, typically with technical triplicates, all with consistent results. The numbers of independent experiments is given and individual results are shown were possible. |
| Randomization | Randomization was not applicable as no experiments involving humans/animals were performed. Importantly, no experiment was sensitive to the order of measurement/treatment. |
| Blinding | Blinding was not carried out as no subjective analysis (e.g. scoring) was performed. |

# Reporting for specific materials, systems and methods

We require information from authors about some types of materials, experimental systems and methods used in many studies. Here, indicate whether each material, system or method listed is relevant to your study. If you are not sure if a list item applies to your research, read the appropriate section before selecting a response.

### Materials & experimental systems

| n/a | Involved in the study | |
|---|---|---|
| ☐ | ☒ | Antibodies |
| ☐ | ☒ | Eukaryotic cell lines |
| ☒ | ☐ | Palaeontology and archaeology |
| ☒ | ☐ | Animals and other organisms |
| ☒ | ☐ | Clinical data |
| ☒ | ☐ | Dual use research of concern |
| ☒ | ☐ | Plants |

### Methods

| n/a | Involved in the study | |
|---|---|---|
| ☒ | ☐ | ChIP-seq |
| ☒ | ☐ | Flow cytometry |
| ☒ | ☐ | MRI-based neuroimaging |

# Antibodies

| | |
|---|---|
| Antibodies used | Primary antibodies:<br>anti-Flag, 1:1000, Sigma, F3165 (clone M2)<br>anti-USP30, 1:500, Sigma, HPA016952 (polyclonal)<br>anti-Vinculin, 1:10000, Sigma, V9131 (clone hVIN-1)<br>anti-GAPDH, 1:10000, Thermo Fisher, AM4300 (clone 6C5)<br>anti-TOM40, 1:2000, Proteintech, 18409-1-AP (polyclonal)<br>anti-TOM20, 1:5000, Proteintech, 11802-1-AP (polyclonal)<br>anti-MFN2, 1:5000, Proteintech, 12186-1-AP (polyclonal)<br><br>Horseradish peroxidase-coupled secondary antibodies:<br>anti-mouse, 1:5000, Sigma, NXA931<br>anti-rabbit, 1:5000, Sigma, GENA934 |
| Validation | All primary antibodies are validated for western blotting on human cell lysates:<br><br>anti-Flag: "Monoclonal ANTI-FLAG® M2 antibody produced in mouse has been used in: immunoblotting. // technique(s): western blot: 10 µg/mL" https://www.sigmaaldrich.com/DE/en/product/sigma/f3165<br><br>anti-USP30: "All Prestige Antibodies Powered by Atlas Antibodies are developed and validated by the Human Protein Atlas (HPA) project and as a result, are supported by the most extensive characterization in the industry. // technique(s): immunoblotting: 0.04-0.4 µg/mL" https://www.sigmaaldrich.com/DE/en/product/sigma/hpa016952<br><br>anti-Vinculin: "Monoclonal Anti-Vinculin antibody produced in mouse has been used in: western blotting. // technique(s): western blot: 1:200 using extract of human fibroblasts" https://www.sigmaaldrich.com/DE/en/product/sigma/v9131<br><br>anti-GAPDH: "Applications: Western Blot (WB), Species Reactivity: Amphibian, Dog, Chicken, Fish, Human, Mouse, Non-human primate, Rabbit, Rat" https://www.thermofisher.com/antibody/product/GAPDH-Antibody-clone-6C5-Monoclonal/AM4300<br><br>anti-TOM40: "KD/KO Validated" "Positive WB detected in HEK-293 cells, HeLa cells" https://www.ptglab.com/products/TOMM40-Antibody-18409-1-AP.htm<br><br>anti-TOM20: "KD/KO Validated" "Positive WB detected in HEK-293 cells, HeLa cells" https://www.ptglab.com/products/TOM20-Antibody-11802-1-AP.htm<br><br>anti-MFN2: "KD/KO Validated" "Positive WB detected in mouse brain tissue, HeLa cells" https://www.ptglab.com/products/MFN2-Antibody-12186-1-AP.htm |

# Eukaryotic cell lines

Policy information about cell lines and Sex and Gender in Research

| | |
|---|---|
| Cell line source(s) | HEK293 cells were purchased from the DSMZ repository (DSMZ no: ACC 305). HeLa cells stably expressing YFP-Parkin were kindly provided by Dr. Richard Youle (National Institute of Neurological Disorders and Stroke, Bethesda, Maryland, U.S.A.). |
| Authentication | Cells were used without authentication. |
| Mycoplasma contamination | Cells were tested for mycoplasma contamination with a negative result. |
| Commonly misidentified lines<br>(See ICLAC register) | No commonly misidentified lines were used in this study. |

# Plants

| | |
|---|---|
| Seed stocks | No plants were involved in this study |
| Novel plant genotypes | No plants were involved in this study |
| Authentication | No plants were involved in this study |
