# Supplementary material for: Chimeric deubiquitinase engineering reveals structural basis for specific inhibition of the mitophagy regulator USP30
Source: Nat Struct Mol Biol. 2025 May 5;32(9):1776–86. doi: 10.1038/s41594-025-01534-4 (PMC12440824; doi:10.1038/s41594-025-01534-4)

Figure 1i

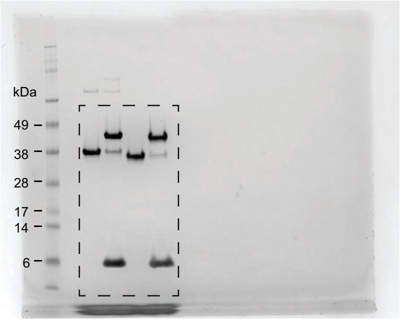

Extended Data Figure 3b

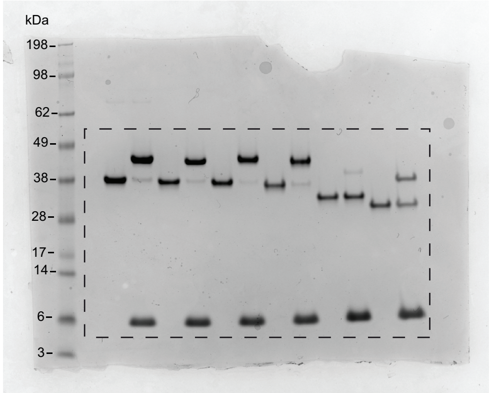

Extended Data Figure 7c

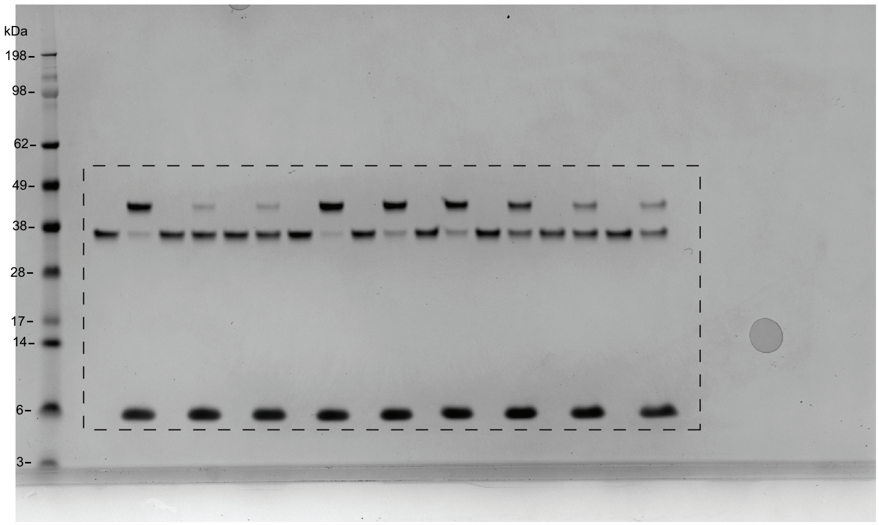

Extended Data Figure 7g

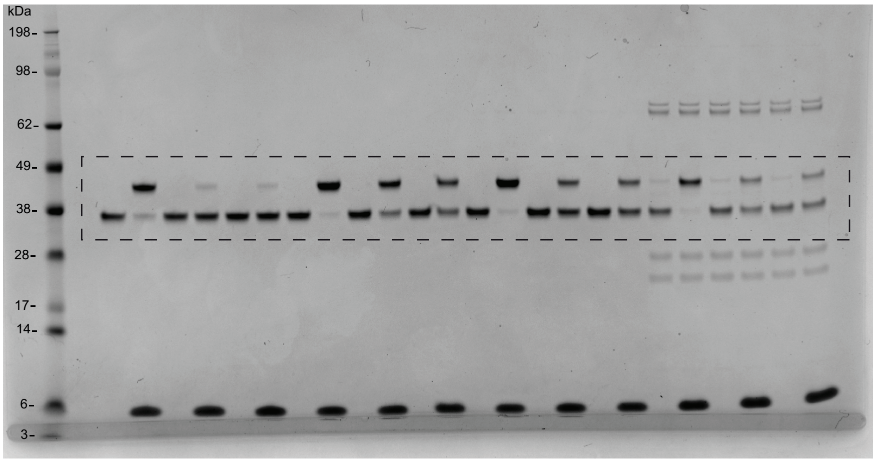

Figure 5a

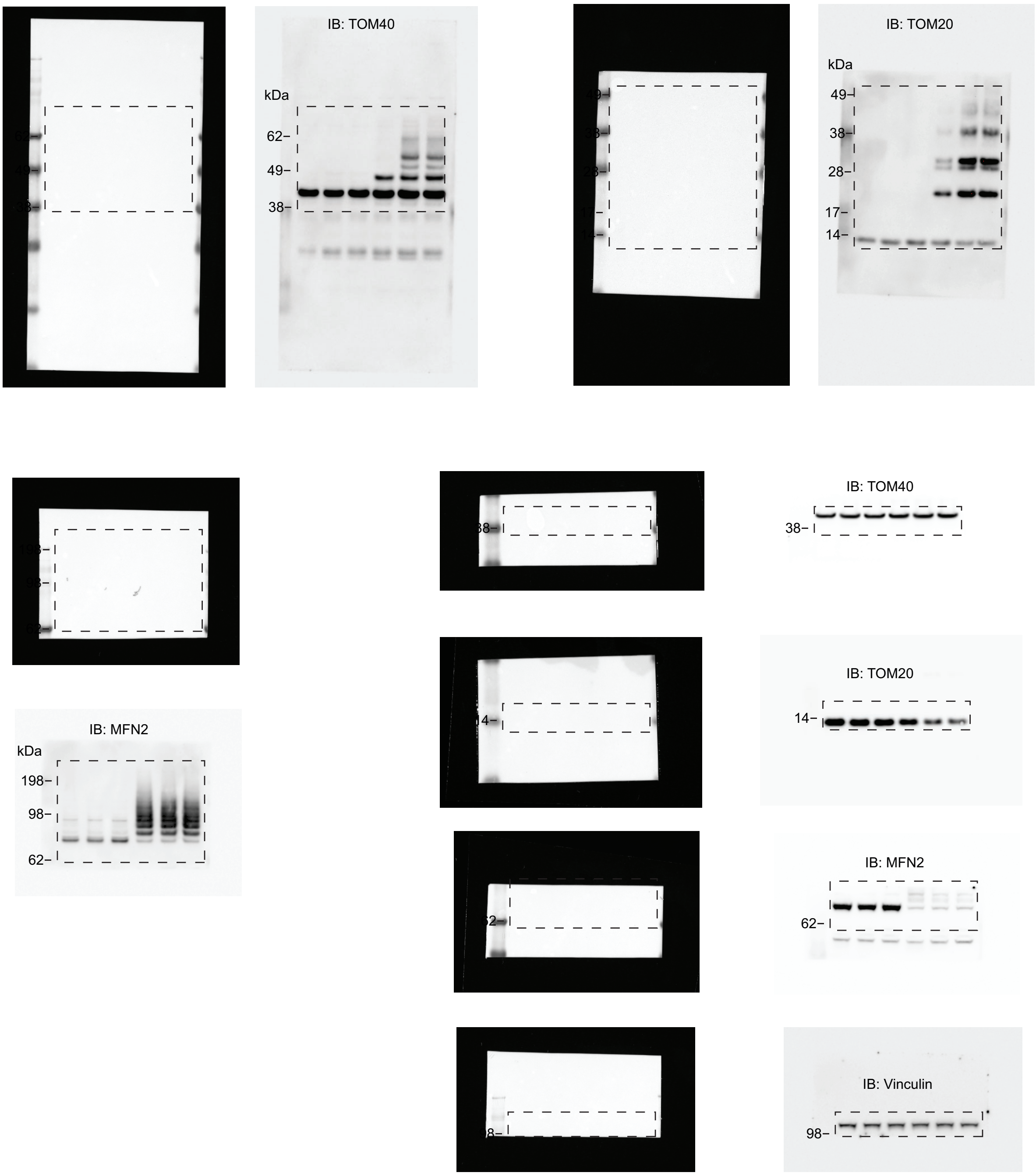

IB: TOM40
kDa
62
49
38
IB: TOM20
kDa
49
38
28
17
14
IB: MFN2
kDa
198
98
62
IB: TOM40
38
IB: TOM20
14
IB: MFN2
62
IB: Vinculin
98

Figure 5b

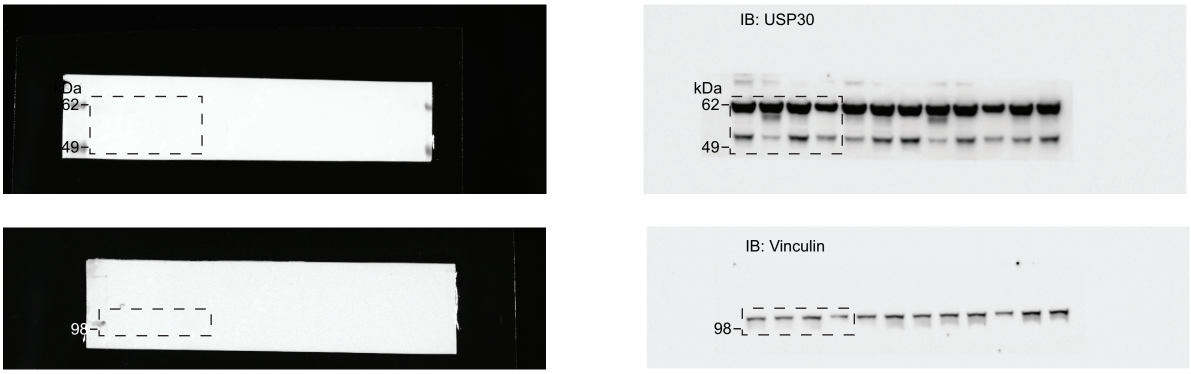

Figure 5c

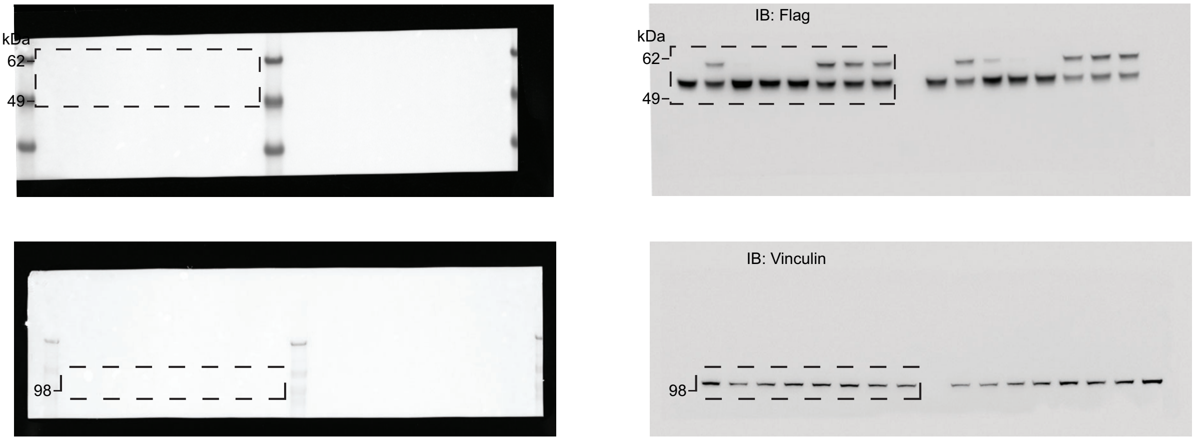

Figure 5f

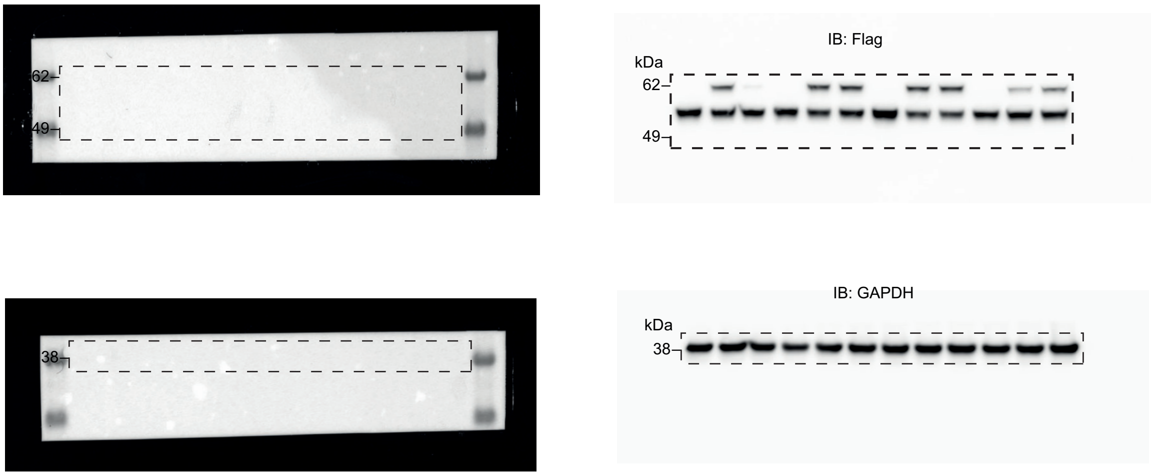

Supplement: Supplementary file 6 — Uncropped gels and blots. [file 41594_2025_1534_MOESM6_ESM.pdf]
